# Lifetime Increased Risk of Adult Onset Atopic Dermatitis in Adolescent and Adult Patients with Food Allergy

**DOI:** 10.3390/ijms18010042

**Published:** 2016-12-27

**Authors:** Hsu-Sheng Yu, Hung-Pin Tu, Chien-Hui Hong, Chih-Hung Lee

**Affiliations:** 1 Department of Food Science, National Pingtung University of Science and Technology, Pingtung 912, Taiwan; bacillus25@gmail.com; 2 Department of Public Health and Environmental Medicine, School of Medicine, College of Medicine, Kaohsiung Medical University, Kaohsiung 807, Taiwan; p915013@cc.kmu.edu.tw; 3 Department of Medical Research, Kaohsiung Medical University Hospital, Kaohsiung 807, Taiwan; 4 Department of Dermatology, National Yang Ming University College of Medicine, Taipei 112, Taiwan; chhong@vghks.gov.tw; 5 Department of Dermatology, Kaohsiung Veterans General Hospital, Kaohsiung 813, Taiwan; 6 Department of Dermatology, Kaohsiung Chang Gung Memorial Hospital and Chang Gung University College of Medicine, Kaohsiung 833, Taiwan

**Keywords:** atopic dermatitis, food allergy, cohort study

## Abstract

Food allergy can result in life-threatening anaphylaxis. Atopic dermatitis (AD) causes intense itching and impaired quality of life. Previous studies have shown that patients with classical early-onset AD tend to develop food allergy and that 10% of adults with food allergies have concomitant AD. However, it is not known whether late-onset food allergy leads to adult-onset AD, a recently recognized disease entity. Using an initial cohort of one-million subjects, this study retrospectively followed-up 2851 patients with food allergy (age > 12 years) for 14 years and compared them with 11,404 matched controls. While 2.8% (81) of the 2851 food allergy patients developed AD, only 2.0% (227) of the 11,404 controls developed AD. Multivariate regression analysis showed that food allergy patients were more likely to develop AD (adjusted hazard ratio = 2.49, *p* < 0.0001). Controls had a 1.99% risk of developing AD, while food allergy patients had a significantly higher risk (7.18% and 3.46% for patients with ≥3 and <3 food allergy claims, respectively) of developing adult-onset AD. This is the first study to describe the chronological and dose-dependent associations between food allergy in adolescence and the development of adult-onset AD.

## 1. Introduction

Atopic dermatitis (AD) is a common allergic disease affecting approximately 10% of the population. The prevalence is increasing, possibly due to modern urbanization and industrialization. AD is usually associated with other allergic diseases, including allergic rhinitis and asthma. The intense itching and skin manifestations in AD often result in insomnia, poor school or work performance, and poor quality of life. The disease pathogenesis is multifactorial, and includes the presence of an abnormal skin barrier and aberrant immune responses. It is well established that AD affects infants or small children, with a fluctuating or progressive course until adulthood. However, following increasing reports of an adult-onset form of the disease, adult-onset AD has recently been recognized as a distinct disease entity, characterized by late disease onset and lesions affecting exposed areas, such as the face and hands. As with adult-onset asthma [1,2], it is believed that adult-onset AD is associated more strongly with environmental factors than the classic AD [3]. Notably, AD in adults was recently reported to be associated with obesity, hypertension, and prediabetes [4], all of which are significant cardiovascular risk factors.

Food allergy is another common allergic disease. Patients with food allergy may develop immediate-type allergy and occasionally suffer from life-threatening anaphylaxis reactions [5]. In the US, approximately 2% of adults and 5% of infants and young children suffer from food allergies, resulting in approximately 30,000 emergency room admissions and 150 individuals’ deaths, per year [6]. Milk, eggs, fish, shellfish, tree nuts, peanuts, wheat, and soybeans account for 90% of food allergies. In Japan, food items containing allergen levels greater than 10 mg/kg must be appropriately labeled, by law. These allergens include eggs, milk, wheat, buckwheat, peanuts, shrimp/prawn, and crab [7]. Taiwan regulations also require food items containing eggs, milk, peanuts, shrimp, crab, and mango, to be labeled [8].

The association between AD and food allergy is not fully understood, although a causal relationship has been suggested [9,10]. Based on population-based studies, the likelihood of food sensitization was reported as approximately six times higher in 3-month-old AD patients compared to healthy controls [11]. Several studies have reported that AD precedes food allergy onset and AD may be associated with sensitization in childhood. Galli et al. reported that AD may precede food allergy [12]. In a cohort by the Danish Allergy Research Centre, 2% of cohort subjects exhibited signs of AD by 3 months of age, while none of the subjects demonstrated symptoms of food allergy. In contrast, one study reported that AD onset at 2 years of age was associated with increased low-level sensitization to eggs, milk, or both, at 6, 12, and 24 months of age (odds ratio (OR) = 1.7–3.3). These results suggest an association between AD and food allergy, although the temporal effect has not been clearly established.

At the population level, approximately 10% of adults with food allergy have concomitant AD. Adult AD patients show much higher rates of food sensitization than controls, particularly to food proteins that are cross-reactive with airborne allergens [13]. These findings are corroborated by an earlier report by our research group, concluding that early and/or current exposure to cigarette smoke (either active or passive) contributes cumulatively to the development of adult-onset AD [14]. However, previous investigations have not evaluated the risk of adult-onset AD among food allergy patients. Using a cohort of one million subjects from the National Health Reimbursement database, which covers 99% of the population in Taiwan, we aimed to determine the association between adult-onset AD in patients with food allergy.

## 2. Results

### 2.1. Demographics of Adult Patients with Food Allergy

**Figure 1:**
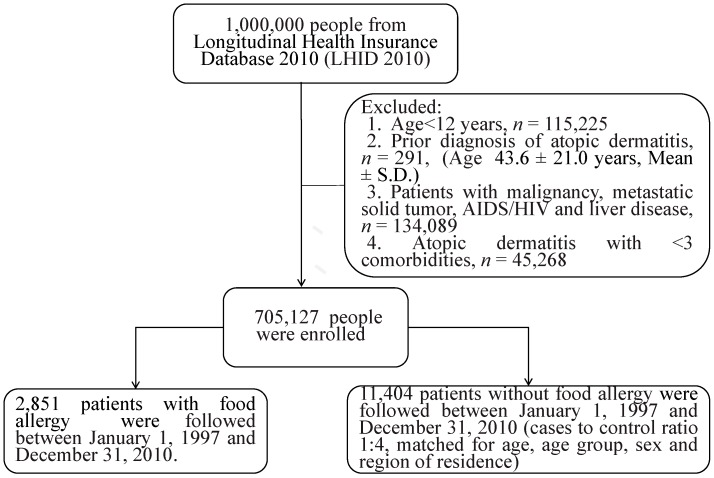
Flow chart of the diagnostic process for food allergy and atopic dermatitis using the Longitudinal Health Insurance Database (LHID) 2010 cohort.

From the initial study cohort of one million subjects, we identified 705,127 subjects (Table 1) for further analysis, based on several exclusion criteria (including young age, cancer, and previous diagnosis of AD; Figure 1). Of these, 2851 (age ≥ 12 years) subjects had a confirmed food allergy diagnosis, and the prevalence of food allergy in our cohort was therefore 0.4%. A high proportion of food allergy patients were aged 20–40 years (1122, 39.4%), and there was a slight female preponderance (female:male = 57.2%:42.8%). Reflecting the higher population density in the northern island, 50.2% of food allergy patients lived in the northern regions. The control group comprised 11,404 matched healthy subjects, and the distribution according to sex, age, and region of residence were similar between the two groups. While 2.8% (81) of the 2851 food allergy patients developed AD, only 2% (227) of the 11,404 matched healthy controls developed AD (*p* = 0.0011). Interestingly, the presence of food allergy was also associated with a higher risk of developing congestive heart failure, chronic pulmonary disease, rheumatoid disease, and renal disease (odds ratio < 2). However, the risk of myocardial infarction, cerebrovascular disease, and diabetes was similar between the two groups.

### 2.2. Food Allergy Patients Have a Significantly Higher Risk of Developing Atopic Dermatitis (AD)

Univariate analysis (Table 2) revealed a potential association between AD and food allergy. To further investigate this finding, we performed a Poisson regression analysis to measure the incidence ratio (IRR) (per 1000 person-years), and a Cox proportional-hazards regression analysis to adjust for potential confounders (age, sex, region of residence, and comorbidities, including congestive heart failure, chronic pulmonary disease, rheumatologic disease, diabetes mellitus, and renal disease). Both male and female food allergy patients had an increased risk of developing AD (male, adjusted hazard ratio (aHR) = 1.82, *p* = 0.0062; female: aHR = 3.07, *p* < 0.0001). Similarly, in the combined group which included both male and female subjects, food allergy was associated with an increased risk of developing AD (aHR = 2.49, *p* < 0.0001).

We then stratified food allergy patients with a confirmed diagnosis of AD (*n* = 81) based on the number of food allergy insurance claims during the study period (claims < 2: *n* = 65; claims ≥ 3: *n* = 16). Patients with a higher number of claims (indicated by a greater number of physician appointments due to food allergies) had a higher risk of developing AD (aHR = 5.13, *p* < 0.0001). This increase in IRR was significantly different between food allergy patients with ≥3 claims and those with 1–2 claims (≥3 claims: 1.67–2.95; 1–2 claims: 3.04–8.63), as indicated by the non-overlapping CIs.

### 2.3. Lifetime Risk of Food Allergy Patients (>12 Years) to Develop Adult AD

**Figure 2:**
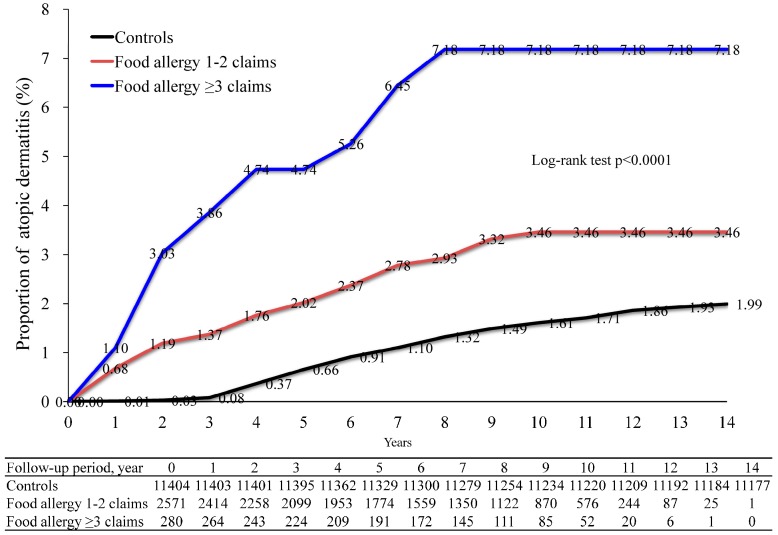
Cumulative incidence of atopic dermatitis in food allergy patients. The incidence of atopic dermatitis in food allergy patients and controls was compared using the log-rank test by a Kaplan–Meier analysis. Food allergy 1–2 claims versus controls, log-rank test *p* < 0.0001; Food allergy ≥3 claims versus controls, log-rank test *p* < 0.0001; Food allergy ≥3 claims versus food allergy 1–2 claims, log-rank test *p* = 0.0053

We demonstrated an overall increase in the risk of AD development in food allergy patients (Figure 2). We investigated the cumulative incidence of AD in these patients (food allergy patients with ≥3 and 1–2 claims, and healthy controls) after several years of follow-up, using the log-rank test with a Kaplan–Meier analysis (Figure 2). After 14 years of follow-up, the baseline risk of AD development in controls (>12 years of age) was 1.99%. Conversely, patients with 1–2 food allergy claims had a significantly higher AD development risk (3.46%, *p* < 0.0001). Moreover, patients with ≥3 claims of food allergy had an even higher AD development risk (7.18%, *p* < 0.0001).

### 2.4. Adolescents with Food Allergy Are Associated with AD According to Joint Effects of Food Allergy and Age Group

To further investigate the interactions between the development of AD and the age of occurrence and presence of food allergies, we calculated the adjusted relative risks (aRR) using a Cox proportional-hazards regression model, after adjustment for age group, sex, the region of residence, and comorbidities (Table 3). An aRR = 1 was defined for the 20–40-year-old (reference age group) control subjects without food allergy. Consistent with previous knowledge, healthy adolescents (12–20-years) had a higher risk of developing AD (aRR = 1.90) than healthy adults (20–40-years). In the reference age group (20–40-years), the presence (aRR = 1.21) or absence (aRR = 1) of food allergy did not affect the risk of developing AD, while this risk was significantly higher in the other age groups (12–20-years: aRR = 2.61, *p* = 0.0008; 40–60-years: aRR = 1.59, *p* = 0.0489).

## 3. Discussion

In this study, we observed that adolescent patients (>12 years) with food allergies have an increased risk of developing AD. Starting from a cohort of one million subjects, AD developed in 2.8% of patients with food allergy and in only 2% of healthy control subjects. The cumulative 14-year incidence rate of AD in patients with ≥3 food allergy claims was 7.18%, in contrast to the baseline 1.99% rate in controls. Interestingly, the presence of early onset food allergy (between 12 and 20 years of age) was associated with AD in the analysis of the combined effect of age and the presence of food allergy.

In this large-scale nationwide cohort study, we retrospectively followed-up patients with food allergy for 14 years. This is the first study demonstrating that adult and adolescent patients with food allergies have an increased risk of developing AD, specifically patients with ≥3 insurance claims for food allergies. We report a time-dynamic increase in the lifetime risk for patients with food allergies to develop AD. In a meta-analysis of 15 studies, food allergy-afflicted children (4–8-years) had an increased risk (OR = 2–3) of developing asthma, AD, and allergic rhinitis [15]. However, further validation is warranted, due to potential residual confounders in the majority of these studies and the lack of follow-up into adolescence and adulthood [15]. Moreover, the risk of AD development in adolescent patients with food allergy has not been reported previously.

AD is known to precede food allergy in many cases, as the impaired skin barrier allows the environmental food allergens to penetrate the skin, leading to systemic allergen sensitization [16]. In a recent study, an impaired skin barrier and filaggrin mutations in AD were implicated in the sensitization to food allergens in children [17]. However, the reason for the increased risk of AD among adolescent and adult patients with food allergy remains unclear. It is possible that food allergy might initiate late-onset AD due to food allergen sensitization in the gut, followed by subsequent skin inflammation.

The two diseases are closely associated. Food allergens provoke AD in 35% of AD patients, as demonstrated in double-blind placebo-controlled food challenge studies [18]. Considering the distinct characteristics and manifestations of adult-onset AD and late-onset food allergy, compared to classical AD and childhood food allergy, further investigations on the risk, among adolescent and adult patients with food allergy, of developing adult-onset AD is warranted. Population studies have shown that approximately 10% of adults with food allergies have concomitant AD and that adult AD patients have higher rates of sensitization to foods than healthy individuals [19]. Shellfish and fish are common sensitization allergens in adult-onset AD, in addition to the common allergens for children [20]. This is consistent with a retrospective study of 369 food allergy-related emergency room admissions (average age, 32.9 years) at a medical center in Taiwan, with over two-thirds of admissions attributed to seafood allergy [21]. Therefore, although adult food allergy is not as common as food allergy in children, the development, as well as exacerbation of adult-onset AD in patients with food allergy, particularly seafood allergy, should be acknowledged.

In the present study, food allergy patients exhibited higher risks of developing congestive heart failure, myocardial infarction, peripheral vascular disease, chronic pulmonary disease, rheumatoid disease, and renal disease. The release of mediators during allergic insults may induce coronary artery spasm and atheromatous plaque erosion or rupture, namely, the Kounis syndrome, characterized by the concurrence of acute coronary syndromes with mast cell activation and anaphylactic insults [22]. A recent study found that adults with AD may have an increased incidence of cardiovascular disease, heart attack, and stroke [23]. Many reports have indicated a strong association between idiopathic nephrotic syndrome and allergic diseases. It is possible that IL-13, a known stimulator of the IgE response, may mediate proteinuria in patients with minimal change disease by directly inducing CD80 expression in the podocyte [24]. Interestingly, one study reported that young children with severe liver dysfunction have a high prevalence of food sensitization [25], indicating that food allergy may be a systemic disease, and not merely an allergic disease or a form of food intolerance. Although the underlying mechanism is unclear, it is possible that the chances of diagnosing chronic pulmonary disease and rheumatoid disease are higher in patients with food allergy, due to the concomitant involvement of rheumatologists, dermatologists, and allergologists, in their management. Food allergies and AD belong to a spectrum of allergic diseases with Th2 polarization. However, other diseases, such as renal diseases, may not be associated with Th2 polarization. We speculated that common epitopes in the digestive system and skin may be involved in the shared Th2 responses in food allergy and/or AD.

There are limitations to this study. First, as this study is based on the ICD-9 diagnosis codes obtained from a reimbursement-purposed health insurance database, the information on specific food allergens was unavailable. A future multicenter registry system-based study, incorporating outpatient clinics and emergency units, may be helpful. Second, the diagnosis of food allergy may vary, according to the experiences of the attending physicians. A diagnosis of food allergy should be based on clinical history, skin prick or blood screening test, followed by an elimination diet and/or standardized oral food challenge [26]. Third, as our data is drawn from an insurance database, it is difficult to analyze the clinical effects of food prevention and elimination to further consolidate the hypothesis that food allergy drives AD development. Potential risk factors, such as lifestyle, nutrition, and physical activity, are not routinely recorded in the National Health Insurance Research Database (NHIRD; www.nhri.org.tw/nhird/). Fourth, based on the study design, AD might be present before the age of 12 years (*n* = 59). However, when we excluded these 59 cases, the association between AD and food allergy was sustained (data not shown). Fifth, because AD runs a recurrent course, AD reappearing after a period of spontaneous remission may be coded as a new case of AD. However, as the recurrence/remission may equally affect patients with or without food allergy, this association may be comparable between the two groups.

In summary, we reported that adolescent patients with food allergy (12–20 years of age) have an increased risk of developing AD. The cumulative 14-year incidence rate of AD in patients with ≥3 food allergy-related insurance claims is 7.18%. Patients with early onset food allergy (12–20-years) are particularly at risk of developing AD. These findings indicate that early food allergy (in patients between 12 and 20 years old) is associated with the development of adult AD.

## 4. Experimental Section

### 4.1. The Database

The National Health Research Institutes in Taiwan governs the use of the National Health Insurance Research Database (NHIRD), which is one of the largest nationwide population-based databases. A single government insurance bureau covers over 99% of all health insurance claims of the population. The database, including information on patient demographics, diagnoses, and prescriptions during inpatient and outpatient services, is available to researchers, after a double-scrambling protocol that masks the original identifiable information to protect privacy. For this study, we utilized the NHIRD Longitudinal Health Insurance Database (LHID) 2010, a nationally representative group of one million insurance beneficiaries who enrolled in the NHIRD in 2010, randomly sampled from the 2010 Registry for Beneficiaries of the NHIRD. As the NHI program was initiated in 1996, the claims data for 1996 were incomplete, and therefore, we included data from individuals enrolled between 1 January 1997 and 31 December 2010.

### 4.2. Study Participants

The selection process of the study population is shown in Figure 1. All diagnoses were based on the International Classification of Diseases, Ninth Revision, Clinical Modification (ICD-9-CM) codes. From this nationally representative sample of one million subjects, we identified 2851 patients with food allergies (ICD-9-CM code 693.1). A total of 11,404 control subjects (at a ratio of one case to four controls) were identified and matched for age, age group, sex, and region of residence. We identified patients aged ≥12 years who were alive on 31 December 2010 to constitute the present study cohort. Patients over 100 years of age were excluded. The outcome of interest in our study was the documentation of AD (ICD-9-CM code 691.8) by a physician in the outpatient/inpatient claims. To enhance the accuracy of outcome identification, we confirmed the diagnosis of AD by identifying at least three outpatient/inpatient attendances in the diagnosis records and excluded patients with <3 outpatient/inpatient attendances. Importantly, we excluded patients who developed AD before the diagnosis of a food allergy.

### 4.3. Co-Morbidities and Confounders

In addition to the demographic risk factors (age, sex, and region of residence), we evaluated other potential confounding factors for myocardial infarction (410.x, 412.x), congestive heart failure (398.91, 402.01, 402.11, 402.91, 404.01, 404.03, 404.11, 404.13, 404.91, 404.93, 425.4–425.9, 428.x), peripheral vascular disease (093.0, 437.3, 440.x, 441.x, 443.1–443.9, 447.1, 557.1, 557.9, V43.4), cerebrovascular disease (362.34, 430.x–438.x), chronic pulmonary disease (416.8, 416.9, 490.x–505.x, 506.4, 508.1, 508.8), rheumatologic disease (446.5, 710.0–710.4, 714.0–714.2, 714.8, 725.x), diabetes mellitus (without chronic complication 250.0–250.3, 250.8, 250.9; and with chronic complication 250.4–250.7), and renal disease (403.01, 403.11, 403.91, 404.02, 404.03, 404.12, 404.13, 404.92, 404.93, 582.x, 583.0–583.7, 585.x, 586.x, 588.0, V42.0, V45.1, V56.x). We excluded comorbidities associated with allergic diseases, including any malignancy (lymphoma and leukemia, except malignant neoplasm of the skin 140.x–172.x, 174.x–195.8, 200.x–208.x, 238.6), metastatic solid tumor (196.x–199.x), AIDS/HIV (042.x–044.x), liver disease (mild 070.22, 070.23, 070.32, 070.33, 070.44, 070.54, 070.6, 070.9, 570.x, 571.x, 573.3, 573.4, 573.8, 573.9, V42.7; and moderate or severe 456.0–456.2, 572.2–572.8). A comorbidity was defined as ≥3 outpatient claims.

### 4.4. Statistical Analysis

We performed a propensity analysis through logistic regression to obtain a 5-digit match of the propensity score for each patient, with age, age group, sex, and the residential region, as covariates. Continuous and categorical variables (demographic characteristics of the study population) were analyzed using the *t*-test and chi-square test, respectively, for comparison between the food allergy and control groups. The incident rate ratio (IRR) was calculated using a generalized linear model (PROC GENMOD) to perform a Poisson regression analysis (a log-linear model). The hazard ratios (HRs) and 95% confidence intervals (CI) for AD were calculated using the Cox proportional hazards model. The Kaplan–Meier method was used to estimate survival curves for each group, and the log-rank test was used to test the homogeneity between survival curves. Age group and region of residence, for the sex-specific groups, and sex and region of residence, for the combined group, were used as matching variables in the STRATA statement, a SAS procedure. Thus, each unique age group and region for the sex-specific groups, and sex and region for the combined group, defined a stratum. The relative risk (RR) of AD events according to the combined effects of food allergy and age group was calculated using a Cox proportional hazards model. Potential risk factors, including age group, sex, region of residence, and comorbidities (congestive heart failure, chronic pulmonary disease, rheumatologic disease, diabetes mellitus, or renal disease), were incorporated into the model. All statistical analyses were performed using the SAS statistical software, version 9.4 (SAS Institute, Cary, NC, USA). The significance level was set at 0.05 with two tails.

## Figures and Tables

**Table 1:** Characteristics of subjects with food allergy and matched controls (1:4).

Group	Food Allergy	Controls	*p*-Value
*N*	2851	11,404	
Atopic dermatitis (691.8), *n* (%)	81 (2.8)	227 (2.0)	0.0052
Duration, *n* (%)			
≤3 years	20 (0.7)	32 (0.3)	
>3 years	61 (2.1)	195 (1.7)	0.0011
Age, Mean (SD), years	41.5 (18.1)	41.4 (17.9)	0.8448
Group, *n* (%)			
12 to <20 years	341 (12.0)	1364 (12.0)	
20 to <40 years	1122 (39.4)	4500 (39.5)	
40 to <60 years	925 (32.4)	3716 (32.6)	
≥60 years	463 (16.2)	1824 (16.0)	0.9911
Sex, *n* (%)			
Male	1219 (42.8)	4797 (42.1)	
Female	1632 (57.2)	6607 (57.9)	0.5029
Region, *n* (%)			
Northern	1432 (50.2)	5491 (48.1)	
Central	576 (20.2)	2599 (22.8)	
Southern	716 (25.1)	2951 (25.9)	
Eastern	94 (3.3)	270 (2.4)	
Offshore islets and other	33 (1.2)	93 (0.8)	0.9824
Comorbidities, *n* (%)			
Myocardial infarction	5 (0.2)	40 (0.4)	0.1354
Congestive heart failure	88 (3.1)	212 (1.9)	<0.0001
Peripheral vascular disease	79 (2.8)	183 (1.6)	<0.0001
Cerebrovascular disease	132 (4.6)	490 (4.3)	0.4360
Chronic pulmonary disease	534 (18.7)	1484 (13.0)	<0.0001
Rheumatologic disease	68 (2.4)	189 (1.7)	0.0090
Diabetes mellitus	189 (6.6)	694 (6.1)	0.2814
Renal disease	56 (2.0)	146 (1.3)	0.0057

SD: standard deviation; Comorbidities defined as ≥3 recorded comorbidities on outpatient claims. Continuous and categorical variables were compared using the *t*-test and *χ*-square test, respectively.

**Table 2:** Adult food allergy patients are at risk of developing atopic dermatitis.

Group	Atopic Dermatitis/Total Subjects (%)	Person-Years at Risk	Incidence Rate Per 1000 Person-Years (95% CI)	IRR (95% CI)	*p*-Value	Adjusted HR (95% CI)	*p*-Value
**Male Cohort Set**							
Controls	101/4797 (2.11)	66,464	1.52 (1.51–1.53)	1.00		1.00	
FA	29/1219 (2.38)	8463	3.43 (3.35–3.50)	2.26 (1.49–3.41)	0.0001	1.82 (1.19–2.81)	0.0062
**Female Cohort Set**							
Controls	126/6607 (1.91)	91,600	1.38 (1.37–1.38)	1.00		1.00	
FA	52/1632 (3.19)	11,001	4.73 (4.64–4.82)	3.44 (2.49–4.75)	<0.0001	3.07 (2.19–4.31)	<0.0001
**Model 1: Combined Group**							
Controls	227/11,404 (1.99)	158,064	1.44 (1.43–1.44)	1.00		1.00	
FA	81/2851 (2.84)	19,463	4.16 (4.10–4.22)	2.90 (2.25–3.73)	<0.0001	2.49 (1.91–3.25)	<0.0001
**Model 2: Combined Group**							
Controls	227/11,404 (1.99)	158,064	1.44 (1.43–1.44)	1.00		1.00	
FA 1–2 claims	65/2571(2.53)	17,604	3.69 (3.64–3.75)	2.57 (1.95–3.39)	<0.0001	2.22 (1.67–2.95)	<0.0001
FA ≥ 3 claims	16/280 (5.71)	1859	8.60 (8.22–9.01)	5.99 (3.61–9.95)	<0.0001	5.13 (3.04–8.63)	<0.0001

FA: food allergy. Incident rate ratio (IRR) was calculated using a SAS procedure (PROC GENMOD) to perform a Poisson regression analysis (a log-linear model). Hazard ratios (HRs) with 95% confidence intervals (CI) and their *p*-values were calculated and adjusted for comorbidities (congestive heart failure, chronic pulmonary disease, rheumatologic disease, diabetes mellitus, renal disease) using a Cox proportional-hazards regression model.

**Table 3:** Early food allergy in adolescents is associated with Atopic dermatitis (AD) according to combined effects of food allergy and age group.

FA	Age Group	Atopic Dermatitis Events, *n* (%)	Total	RR (95% CI)	*p*-Value	Adjusted RR (95% CI)	*p*-Value
No	12 to <20	44 (3.23)	1364	2.08 (1.42–3.04)	0.0002	1.90 (1.29–2.79)	0.0011
No	20 to <40	71 (1.58)	4500	1.00		1.00	
No	40 to <60	64 (1.72)	3716	1.09 (0.78–1.54)	0.6082	1.03 (0.73–1.45)	0.8779
No	≥60	48 (2.63)	1824	1.69 (1.16–2.44)	0.0057	1.22 (0.82–1.82)	0.3201
Yes	12 to <20	16 (4.69)	341	3.07 (1.77–5.35)	<0.0001	2.61 (1.49–4.58)	0.0008
Yes	20 to <40	22 (1.96)	1122	1.25 (0.77–2.02)	0.3691	1.21 (0.75–1.97)	0.4343
Yes	40 to <60	26 (2.81)	925	1.80 (1.14–2.84)	0.0110	1.59 (1.00–2.51)	0.0489
Yes	≥60	17 (3.67)	463	2.38 (1.39–4.07)	0.0016	1.49 (0.84–2.65)	0.1763

FA: food allergy. Relative risk (RR) with 95% confidence intervals (CI) and their *p*-values were calculated using a Cox proportional-hazards regression model. Adjusted RR was calculated and adjusted for age group, sex, region, and comorbidities using a Cox proportional-hazards regression model.
